# Fabrication and Evaluation of Gellan Gum/Hyaluronic Acid Hydrogel for Retinal Tissue Engineering Biomaterial and the Influence of Substrate Stress Relaxation on Retinal Pigment Epithelial Cells

**DOI:** 10.3390/molecules27175512

**Published:** 2022-08-27

**Authors:** Jina Youn, Joo Hee Choi, Sumi Lee, Wonchan Lee, Seong Won Lee, Wooyoup Kim, Youngeun Song, Nomin-Erdene Tumursukh, Jeong Eun Song, Gilson Khang

**Affiliations:** 1 Department of Bionanotechnology and Bio-Convergence Engineering, Jeonbuk National University, Jeonju-si 54896, Jeonbuk, Korea; 2 Department of PolymerNano Science & Technology and Polymer Materials Fusion Research Center, Jeonju-si 54896, Jeonbuk, Korea; 3 Department of Orthopaedic & Traumatology, Airlangga University, Surabaya 60115, Jawa Timur, Indonesia

**Keywords:** gellan gum, hyaluronic acid, hydrogel, substrate stress relaxation, retinal pigment epithelial cells

## Abstract

Cell therapies for age-related macular degeneration (AMD) treatment have been developed by integrating hydrogel-based biomaterials. Until now, cell activity has been observed only in terms of the modulus of the hydrogel. In addition, cell behavior has only been observed in the 2D environment of the hydrogel and the 3D matrix. As time-dependent stress relaxation is considered a significant mechanical cue for the control of cellular activities, it is important to optimize hydrogels for retinal tissue engineering (TE) by applying this viewpoint. Herein, a gellan Gum (GG)/Hyaluronic acid (HA) hydrogel was fabricated using a facile physical crosslinking method. The physicochemical and mechanical properties were controlled by forming a different composition of GG and HA. The characterization was performed by conducting a mass swelling study, a sol fraction study, a weight loss test, a viscosity test, an injection force study, a compression test, and a stress relaxation analysis. The biological activity of the cells encapsulated in 3D constructs was evaluated by conducting a morphological study, a proliferation test, a live/dead analysis, histology, immunofluorescence staining, and a gene expression study to determine the most appropriate material for retinal TE biomaterial. Hydrogels with moderate amounts of HA showed improved physicochemical and mechanical properties suitable for injection into the retina. Moreover, the time-dependent stress relaxation property of the GG/HA hydrogel was enhanced when the appropriate amount of HA was loaded. In addition, the cellular compatibility of the GG/HA hydrogel in in vitro experiments was significantly improved in the fast-relaxing hydrogel. Overall, these results demonstrate the remarkable potential of GG/HA hydrogel as an injectable hydrogel for retinal TE and the importance of the stress relaxation property when designing retinal TE hydrogels. Therefore, we believe that GG/HA hydrogel is a prospective candidate for retinal TE biomaterial.

## 1. Introduction

Age-related macular degeneration (AMD) is the leading cause of blindness worldwide, and in recent years, it has become one of the most common diseases among elderly people [1]. AMD is associated with the destruction of retinal pigment epithelium (RPE) cells and Bruch’s membrane (BrM) [2,3]. The RPE is a monolayer composed of pigmented epithelial cells in contact with the neural retina, and it is involved in physiological visual functions, such as the absorption of light energy and the secretion of immunosuppressive factors [4]. Various treatments are constantly being studied to regenerate damaged or injured RPE. Recent reports suggest that tissue engineering (TE) treatment can be a promising method for RPE regeneration. TE approaches include the delivery of cells in the subretinal space by using various scaffolds. Selecting suitable biomaterials and designing the scaffold is a significant part of TE. Porous scaffolds are effective in fabricating artificial basement membranes for the culture of RPE cells or stem cells. However, transplantation procedures with porous scaffolds are complicated, as they are difficult to handle.

Recently, hydrogels have been attracting a lot of attention, as they can be injected, which resolves the complicated surgical process [5]. Moreover, hydrogels have a similar structure to the natural extracellular matrix (ECM) and contain a large amount of water, which allows for a suitable microenvironment to encapsulate cells, drugs, and biomolecules [6,7,8,9,10]. Examples of hydrogels used in retinal TE include hyaluronic acid (HA) [11,12,13,14,15,16], methyl (MC) [15,16], gellan gum (GG) [17,18,19], alginate (Alg) [2,20,21], agarose (Ag) [22,23], gelatin (Gel) [14,19,24,25], fibrinogen [23,26,27,28], poly (ethylene glycol) (PEG) [18,21,29,30], and poly-l-lysine (PLL) [13,30,31]. 

Although the application of hydrogels in retinal TE continues to develop, exploring suitable mechanical properties for retinal cells has been neglected. Previously reported studies have shown that the modulus and stiffness of the hydrogel affect retinal cells [21,29]. In addition, cell behavior has been confirmed in the 2D environment of the hydrogel and the 3D matrix. As time-dependent stress relaxation is considered a significant mechanical cue for the control of cellular activities, it is important to optimize hydrogels for retinal TE by applying this viewpoint [32,33,34]. 

Among the various hydrogels presented, HA is the most biocompatible and promising material for retinal TE. HA, which can be found in the native ECM and vitreous body, is a linear polysaccharide composed of two repeating units (β-1,2-d-glucuronic acid and β -1,3-*N*-acetyl-d-glucosamine) [35,36]. HA does not contain CD44 but binds it, the receptor for hyaluronan-medicated motility (RHAMM) and intercellular adhesion molecule 1 (ICAM-1), which supports cell attachment and migration. However, pure HA has a high rate of degradation in vitro and in vivo, and poor mechanical properties. Therefore, chemical modification or blending with other types of polymers is necessary to compensate for the fast degradation rate and to enhance the mechanical characteristics of HA. A composite of HA and other types of biomaterials has the advantage of not only improving the mechanical and physicochemical properties but also enhancing the bioactivity of cells. Various types of biomaterials are reported to be effective in fabricating HA composites (e.g., methylcellulose, alginate, silk fibroin, chitosan, gelatin, collagen, and chondroitin sulfate) [37,38]. Among them, the composite of GG and HA is on the rise to be a promising material and is being used in various TE fields (e.g., cartilage TE, vitreous TE, skin TE, bone TE, and disc TE) [39,40]. GG is a linear anionic polysaccharide composed of tetrasaccharide (1,3-β-d-glucose, 1,4-β-d-glucuronic acid, 1,4-β-d-glucose, and 1,4-α-l-rhamnose) repeating units containing a carboxyl side group [41,42]. GG is a thermoreversible gel that forms a random coil at a high temperature and a double helical structure upon cooling. Moreover, GG can be physically crosslinked with the presence of cations (Na^+^, K^+^, Ca^2+^, and Mg^2+^), which provide a stable structure in the physiological environment; an injectable property; and a viscoelastic character, which is reported to be effective for cell viability and proliferation [43,44]. 

**Scheme 1:**
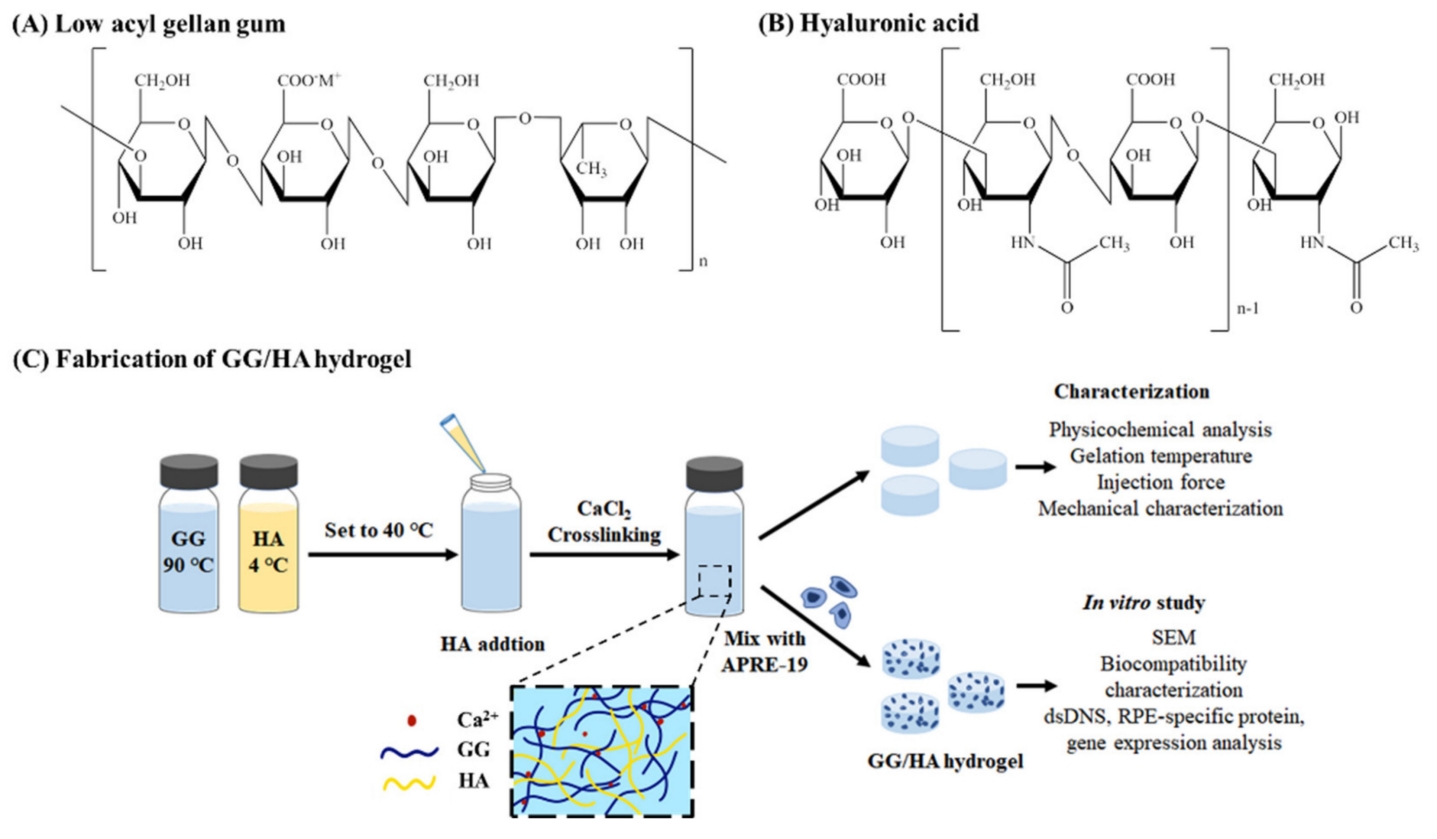
Fabrication process of GG/HA hydrogel. (**A**) Chemical structure of low acyl GG. (**B**) Chemical structure of HA. (**C**) Proposed interactions of GG/HA hydrogel.

Herein, to replace the lost cells as a cell carrier, a GG/HA hydrogel was fabricated using a facile physical crosslinking method. The physicochemical and mechanical properties were controlled by forming different compositions of GG and HA. Characterization was performed by conducting a mass swelling study, a sol fraction study, a weight loss test, a viscosity test, an injection force study, a compression test, and a stress relaxation analysis. The biological activity of the cells encapsulated in 3D constructs was evaluated by conducting a morphological study, a proliferation test, a live/dead analysis, histology, immunofluorescence staining, and a gene expression study to observe the most appropriate material for retinal TE biomaterial. (Scheme 1)

## 2. Results and Discussion

### 2.1. Characterization of GG/HA Hydrogel

#### 2.1.1. Physicochemical Analysis

**Figure 1:**
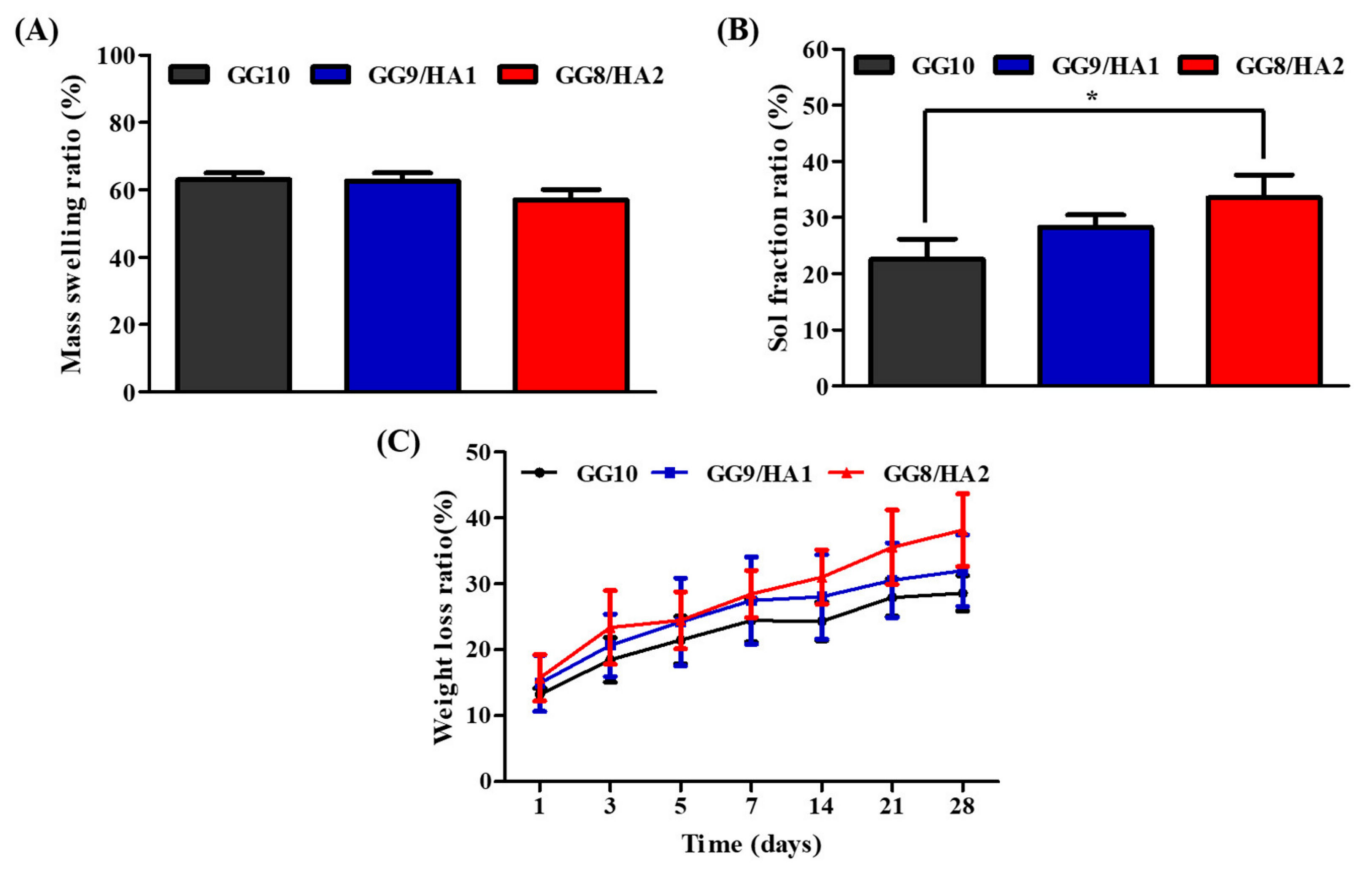
Physicochemical analysis of the hydrogels: (**A**) mass swelling ratio (%), (**B**) sol fraction ratio (%), and (**C**) weight loss ratio (%), analyzed for 28 days (values are means ± SD, *n* = 4, *p* < 0.05 (*)).

Swelling is an important factor to consider when designing a scaffold for TE. It plays a significant role in delivering nutrients and oxygen and in removing residues in vitro and in vivo. The mass swelling of the hydrogels showed a slight reduction, as a higher ratio of HA was contained in the composites (Figure 1A). The mass swelling ratios of the hydrogels were 63.02 ± 2.05, 62.59 ± 2.44, and 57.08 ± 3.04% in the GG10, GG9/HA1, and GG8/HA2 hydrogels, respectively. The swelling occurs from the osmotic pressure of the hydrogel being higher than that of the physiological aqueous environment. Both GG and HA interacted with the ionic component but affected the osmotic pressure due to the reduction of GG, which induced ionic crosslinking. Although the amount of HA increased due to the difference in osmotic pressure, it reduced the water absorption capacity and elastic pressure of the hydrogel [45]. GG hydrogels absorb not only water but also an ionic component in the physiological fluid and form ionic crosslinking. This phenomenon may have increased the elastic pressure of the GG hydrogel, which caused higher swelling. A higher ionic crosslinking density in the matrix was confirmed from the sol fraction ratio study (Figure 1B). The GG10, GG9/HA1, and GG8/HA2 hydrogels displayed a sol fraction of 22.59 ± 3.61, 28.27 ± 2.20, and 33.50 ± 4.06%, respectively. The sol fraction ratio states the percentage of the polymer that is not contained in the crosslinked matrix. Therefore, the higher the sol fraction, the lower the crosslinking density of the composite [46]. It is regarded that the incorporation of HA decreased the bridging effect of Ca^2+^ and the helix chain of GG [39]. The degradation study of the hydrogels was performed for 28 days. The degradation property of the biomaterial is also an important characteristic to consider when fabricating a TE scaffold. The fast degradation rate of the hydrogel may burst and release the encapsulated cells, biomolecules, and drugs in physiological conditions at the initial time point of the treatment. However, a fast decomposition of the biomaterial may not be able to produce a sufficient therapeutic effect or may degrade before the tissue is properly regenerated [39]. The weight loss ratio of the hydrogels was analyzed for 28 days (Figure 1C). Day 1 of the degradation test showed a weight loss of 13.21 ± 0.97, 14.92 ± 4.27, and 15.73 ± 3.52% in GG10, GG9/HA1, and GG8/HA2, respectively. The weight loss of the hydrogels gradually increased as time passed, and on day 28 of culture, GG10, GG9/HA1, and GG8/HA2 exhibited a weight loss of 28.59 ± 2.70, 32.02 ± 5.40, and 38.17 ± 5.54, respectively. The initial rapid weight loss is due to the escape of the polymer that is not involved in crosslinked matrices. The HA-incorporated hydrogels exhibited higher weight loss kinetics due to the higher sol fraction, but they did not show significant differences when compared to GG10. As time passed, the hydrogel gradually degraded, and the GG8/HA2 hydrogel with the lowest crosslinking density showed the highest degradation behavior. 

#### 2.1.2. Mechanical Properties Characterization

**Figure 2:**
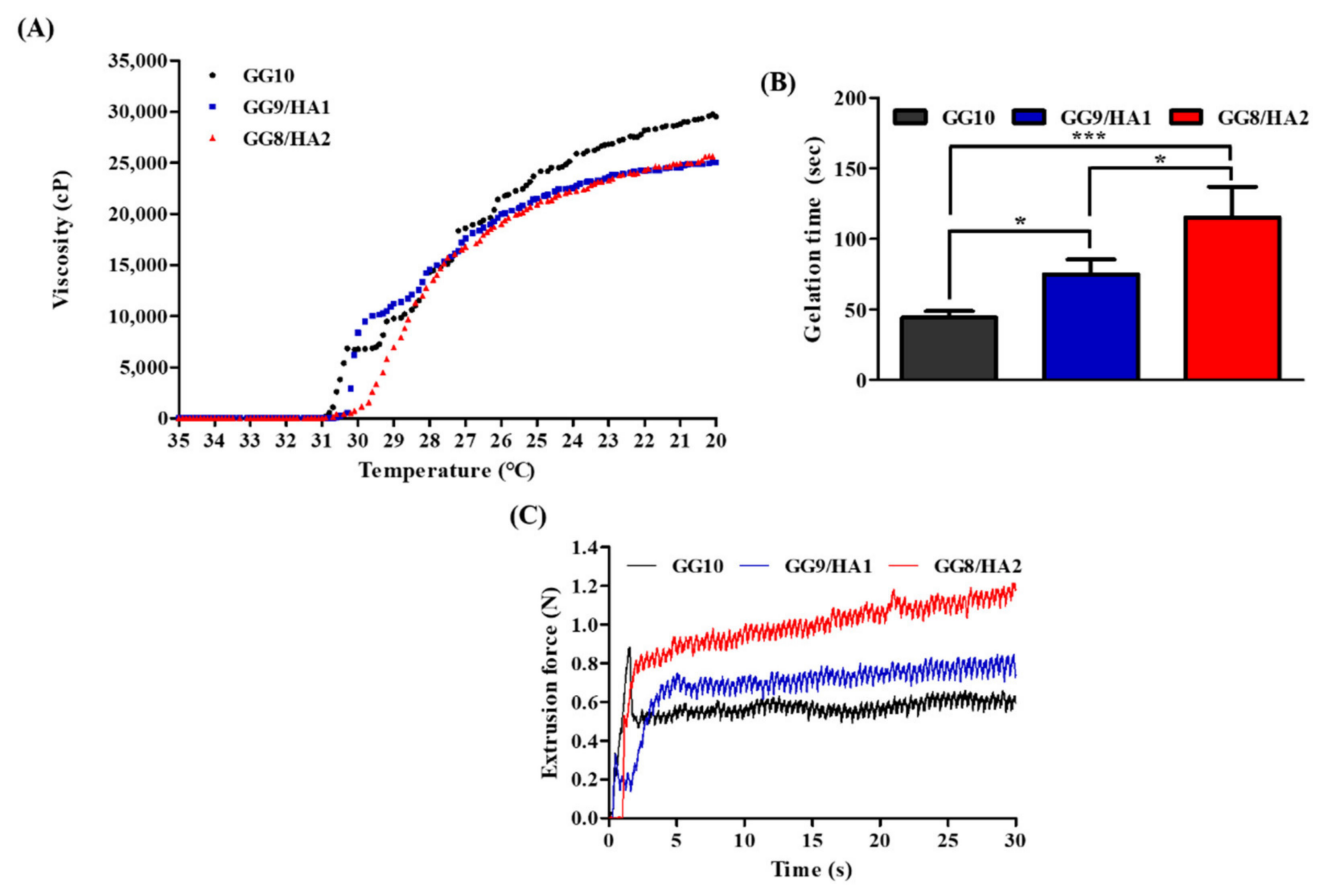
Characterization of gelation temperature, time, and injectability of the hydrogels. (**A**) Viscosity study evaluated at a temperature range of 35 °C to 20 °C; (**B**) analysis of gelation time at the gelation temperature; (**C**) characterization of injection force using a syringe capped with a $26G{\frac{1}{2}}^{\prime \prime }$ needle, Texture Analyzer at a speed of 20 mm/min, and load cell of 20 N (values are mean ± SD (*n* = 4), *p*< 0.05 (*), *p* < 0.001 (***)).

Gelation temperature and gelation time were analyzed to characterize the properties of the fabricated hydrogels (Figure 2). The gelation temperature is important when designing a hydrogel, as a too-high gelation temperature may lead to a high rate of cell death. Moreover, it is important for the gelation of the hydrogel to occur properly in order to evenly disperse cells. The gelation of the GG10, GG9/HA1, and GG8/HA2 hydrogels occurred at 31.35 ± 0.64, 30.45 ± 0.21, and 31.15 ± 0.35°C, respectively (Figure 2A). The gelation temperature was sufficiently low enough for cells to be cultured in the hydrogel solution at a cell-friendly temperature and to solidify at room temperature (RT). The gelation times of the hydrogels were 44.25 ± 4.5, 75.00 ± 10.39, and 115.33 ± 21.55 s (Figure 2B). The lower content of GG and the higher amount of HA inhibited the fast gelation of the matrix due to the lower ionic crosslinking density in the matrix. 

The injectability of the hydrogel was confirmed by the injection force test (Figure 2C). Physically and ionically crosslinked hydrogels have shear-thinning characteristics in which the viscosity decreases under shear strain and self-recover when shear is removed [47]. Thus, this type of hydrogel can be applied as an injectable biomaterial. All the hydrogels displayed injectable properties in the injection test. However, due to the high molecular weight of HA, HA exists as a highly entangled random coil in the overall matrix. Moreover, the lower density of the ionic crosslink reduces the partially aligned matrix, which leads to a lower shear-thinning property. Therefore, the injection force was higher, as a greater amount of HA was incorporated.

**Figure 3:**
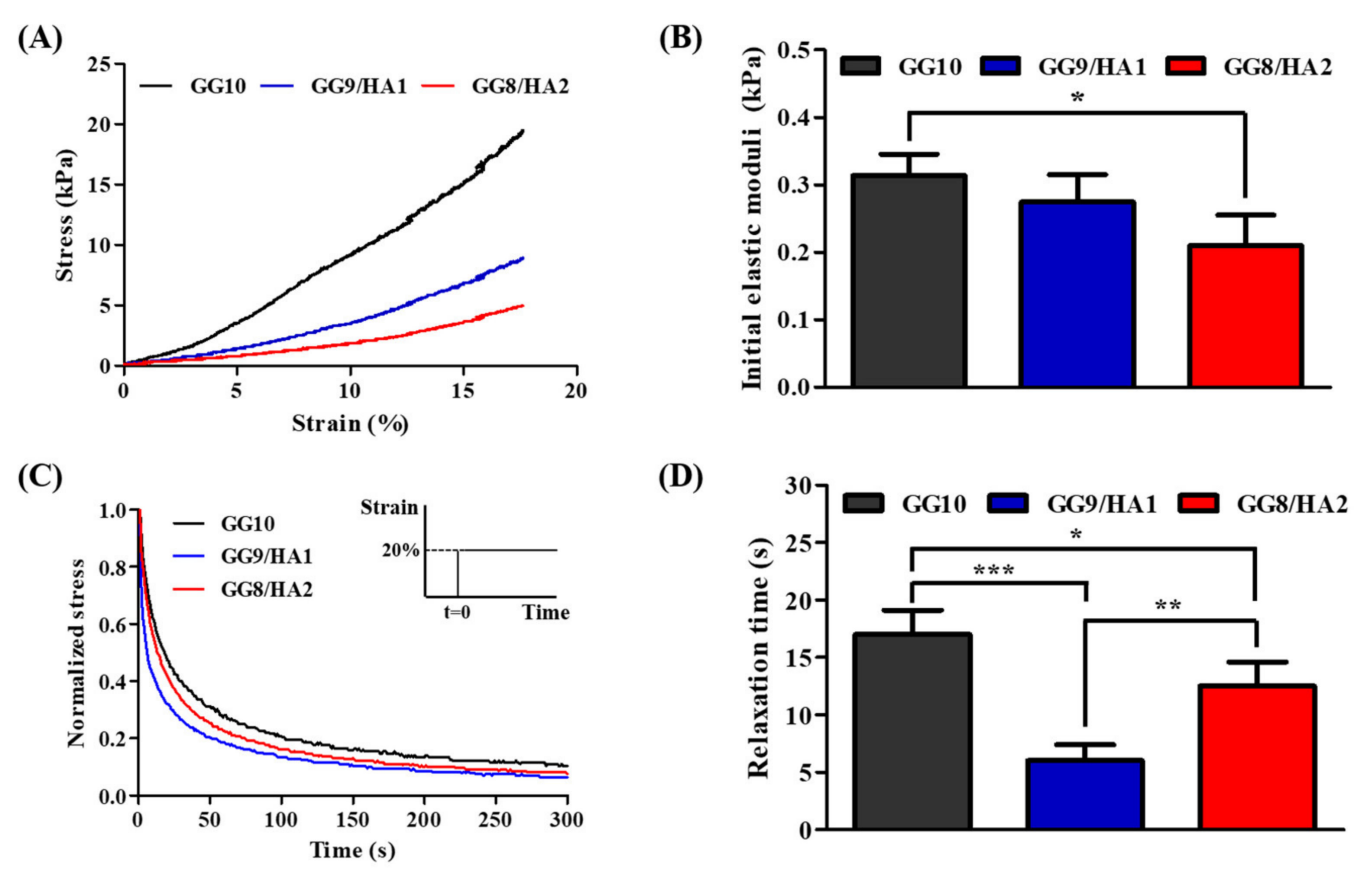
Mechanical characterization of the hydrogels. (**A**) Stress–strain curve of the hydrogels analyzed at a speed of 1 mm/min with a load cell of 10 N; (**B**) initial elastic modulus at 5–10% strain; (**C**) stress relaxation analysis under constant strain; (**D**) normalized relaxation time (values are mean ± SD (*n* = 4), *p* < 0.05 (*), *p* < 0.01 (**), and *p* < 0.001 (***)).

The compressive modulus was characterized to analyze the static mechanical properties of the hydrogels. The elastic modulus of the scaffold has been found to have a great influence on various cell behaviors. A higher elastic modulus promotes cell migration, differentiation, proliferation, and growth [48]. In the compression test, all the hydrogels exhibited suitable mechanical properties to encapsulate cells and to be applied in TE. GG10 exhibited the highest elastic moduli, and the strength gradually decreased as HA was incorporated into the hydrogels (Figure 3A). The initial elastic moduli of the GG10, GG9/HA1, and GG8/HA2 hydrogels were 0.31 ± 0.03, 0.27 ± 0.04, and 0.21 ± 0.05 kPa at 5–10% strain, respectively (Figure 3B). This is due to the higher ionic crosslinking of the pristine GG and the lower crosslinking density as the HA content increased. However, a viscoelastic material exhibits stress relaxation under mechanical loading, and recent studies have powerfully illustrated the significance of the time-dependent aspects of hydrogel mechanics. It has been reported that hydrogels that exhibit faster stress relaxation promote the remodeling matrix, proliferation, and differentiation of cells that are encapsulated in the hydrated 3D network [49]. In the case of the stress relaxation of the fabricated hydrogels all exhibited time-dependent change. Interestingly, compared to the pristine GG, the GG/HA hydrogels exhibited a faster stress relaxation time. The relaxation times at 0.5 of normalized stress were 17.00 ± 2.12, 6.00 ± 1.41, and 12.50 ± 2.12 s in GG10, GG9/HA1, and GG8/HA2, respectively (Figure 3C,D). The unbinding of crosslinks and hydrogel flow leads to relaxation. The pristine GG contained a higher amount of ionic crosslinked density, which lagged the relaxation of the matrix, while the incorporation of HA decreased the ionic crosslinking density and increased the flow of the matrix. However, coinciding with the injection force test, as the content of HA increases, it is suspected that the disordered high-molecular-weight polymers prevent the relaxation of the matrix.

### 2.2. In Vitro Analysis

#### 2.2.1. Morphology Analysis

**Figure 4:**
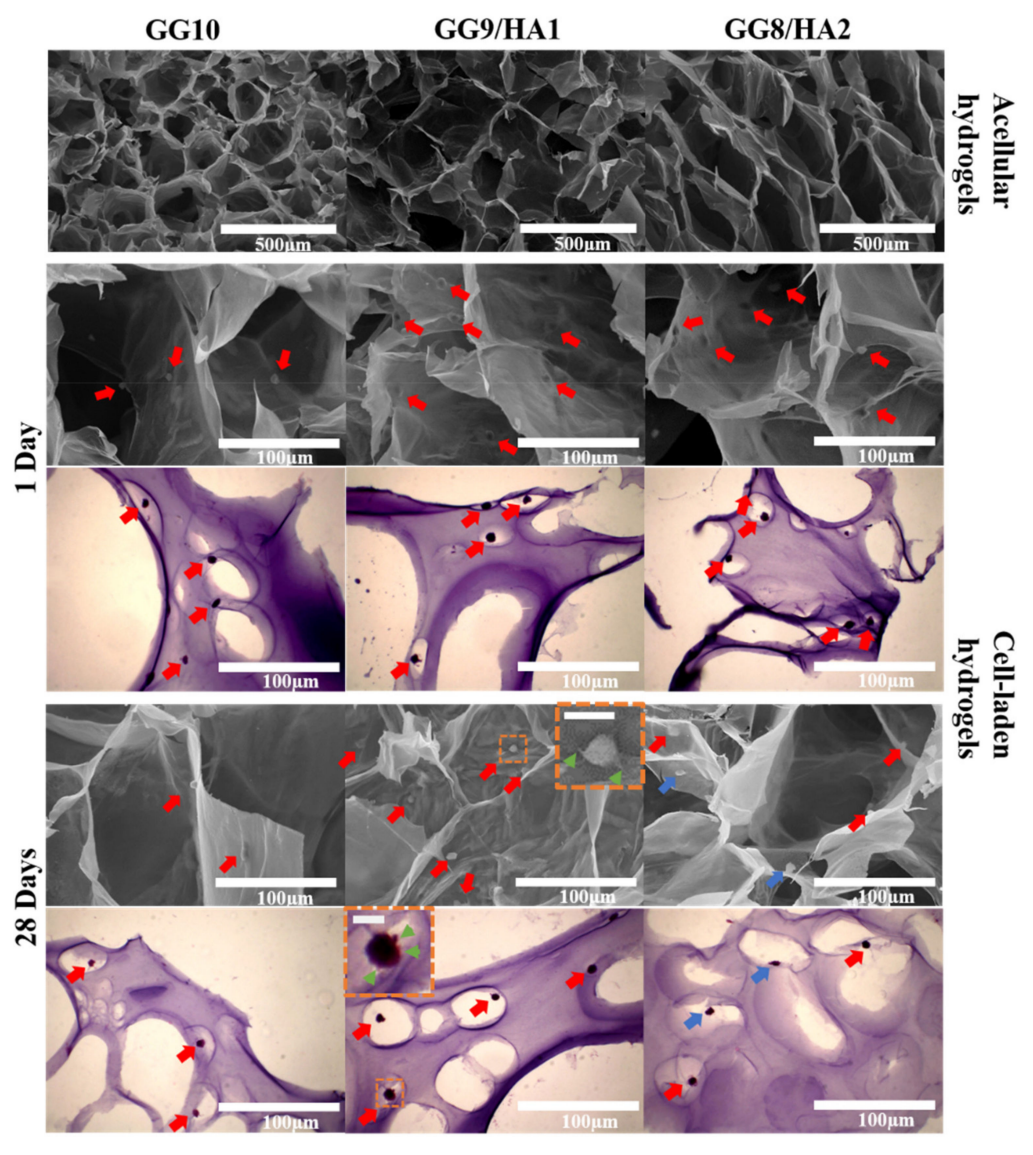
Morphological observation of the acellular and cell-laden hydrogels under SEM analyzed after 1 and 28 days of culture. The suspension of cells in the porous structure (marked in red arrows), enhanced cell–matrix interactions (shown in orange box), secreted extracellular matrix (marked in green arrows), and deformed cell shapes (marked in blue arrows) were observed.

The pore size and porous structure of the acellular and cell-laden hydrogels were observed under SEM and with a histological analysis. All the groups of the lyophilized acellular hydrogels showed a continuous porous structure from the formation of ice crystals caused by a freeze-drying process. The pore size increased as the HA content increased, which may be due to the decreased crosslinking density (Figure 4). The cell-laden hydrogels exhibited cell suspension in the porous structure (marked in red arrows). The encapsulated cells formed a roundel shape, and visibly deformed cells were not observed. A higher number of cells was observed in the HA-loaded hydrogels due to the cell adhesion ability of HA. After 28 days of culture, a higher number of cells was observed in the HA-loaded groups than in the GG group. GG9/HA1 exhibited enhanced cell–matrix interactions (shown in the orange box), which allowed the cells to secrete extracellular matrix (marked in green arrows), while GG8/HA2 showed a rather deformed cell shape (marked in blue arrows). This result shows that, although HA contains cell-binding moieties, appropriate physicochemical properties and degradation rates must be supported to enhance the microenvironment of the hydrogels.

#### 2.2.2. Live/Dead Staining and dsDNA Content Analysis

**Figure 5:**
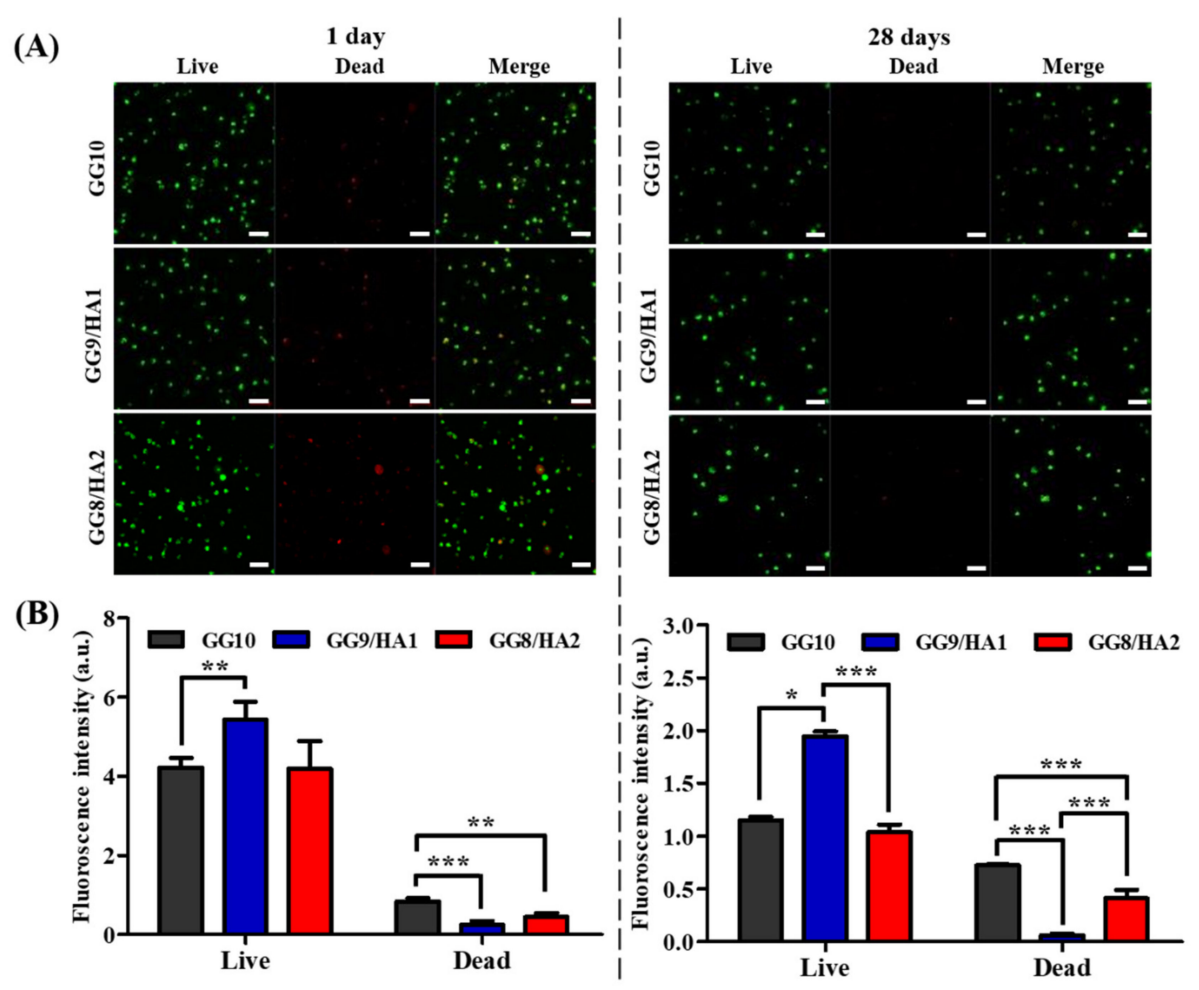
Biocompatibility characterization of the hydrogels: (**A**) live/dead staining images of ARPE-19-encapsulated hydrogels analyzed with the Z-stack mode; (**B**) relative intensity of live/dead images evaluated with ImageJ software (scale bar = 50 μm) (values are mean ± SD, *n* = 4, *p* < 0.05 (*), *p* < 0.01 (**), *p* < 0.001 (***)).

**Figure 6:**
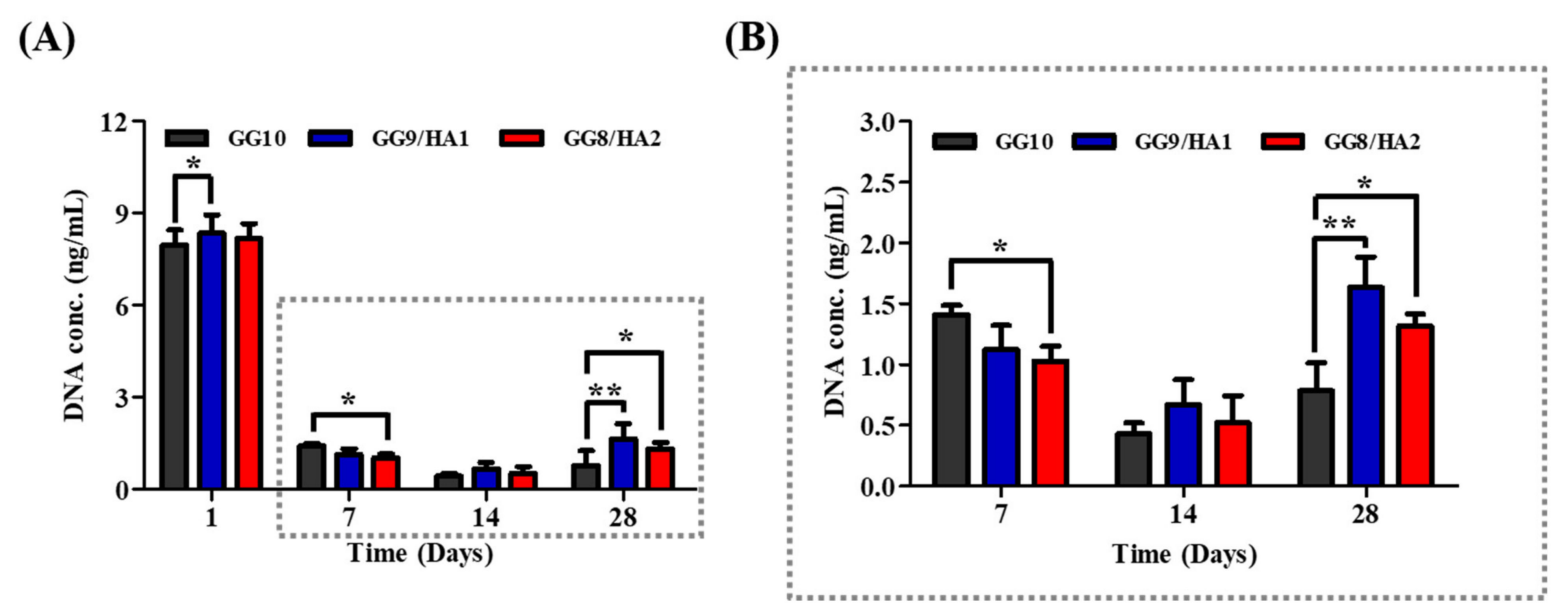
dsDNA content of the APRE-19 encapsulated in the hydrogels: (**A**) analyzed after 1, 7, 14 and 28 days of cell culture, (**B**) enlarged figure of the 7, 14, and 28 days of cell culture (values are means ± SD, *n* = 4, *p* < 0.05 (*), *p* < 0.01 (**)).

The biocompatibility of the constructed hydrogels was analyzed by encapsulating ARPE-19 in the fabricated hydrogels. Calcein AM (green) and ethidium homodimer (red) of the live/dead staining reagent stain live and dead cells, respectively. The cell-laden GG9/HA1 showed a higher intensity of live cells and a lower intensity of dead cells after 1 day of culture than GG10 and GG8/HA2. The live/dead staining of the cell-laden hydrogels cultured for 28 days exhibited a lower number of cells in all the groups than those cultured for 1 day, which is due to the degradation of the composite (Figure 5). The dsDNA content of the cell-laden hydrogels was analyzed after 1, 7, 14, and 28 days of culture to confirm cell proliferation (Figure 6). The dsDNA content on day 1 was 7.95 ± 0.50, 8.33 ± 0.59, and 8.16 ± 0.45 ng/mL in GG, GG9/HA1, and GG8/HA2, respectively. After 7, 14, and 28 days of culture, all the groups showed a significant decrease in the number of cells when compared to that after 1 day of culture, which may be due to the gradual degradation of the composite. The dsDNA content decreased in all the groups until day 14 of culture, and it is suspected that the rate of the decomposition of the matrix was higher than the rate of cell proliferation and growth. After 28 days of culture, the cells exhibited an increased dsDNA content in all the groups. In particular, the highest dsDNA value was observed in GG9/HA1, which coincides with the live/dead results. A previous study showed that the modulus of the hydrogel influences the adhesion, activity, and expression of RPE cells [21,50]. However, in the mechanical analysis of the hydrogels, GG10 showed the highest elastic modulus but poor cell viability and proliferation when compared to the HA-incorporated groups. This suggests that a high modulus does not have a positive effect on ARPE-19 cells in a 3D environment. From another perspective, it is noted that cell migration, spreading, and proliferation, and the deposition of matrix are related to the degradation of the matrix and the viscoelasticity property of the hydrogel. The concept is that the cellular matrix remodeling in a faster stress relaxation 3D matrix overcomes mechanical confinement [34,51,52]. In terms of degradation, GG8/HA2 exhibited the fastest weight loss kinetics, but the viability and proliferation of the cells were lower than those in GG9/HA1. Therefore, it can be suspected that ARPE-19 cells are highly influenced by the rapid stress relaxation of the hydrogels.

#### 2.2.3. RPE-Specific Protein and Gene Expression Study

**Figure 7:**
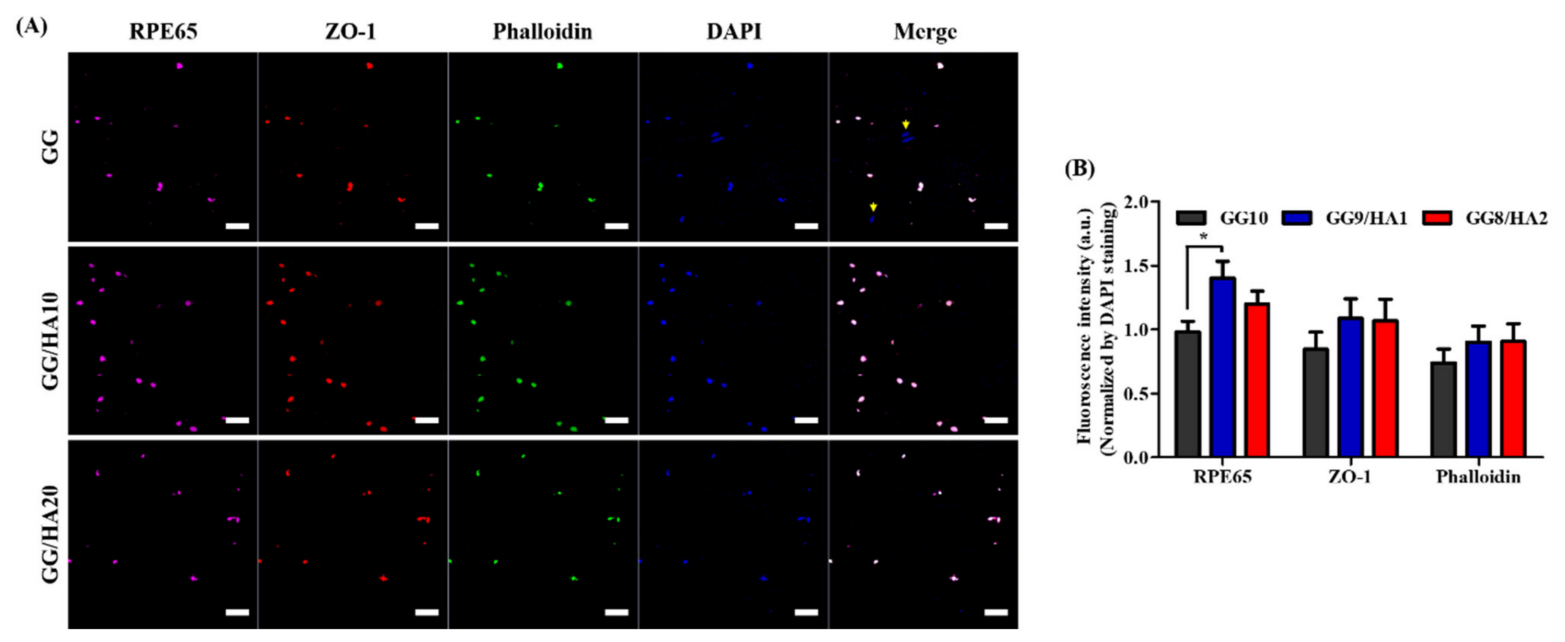
RPE-specific protein expression analyzed by (**A**) immunofluorescence staining of RPE65, ZO-1, phalloidin, and DAPI on 28 days of culture; (**B**) relative intensity of the staining evaluated with ImageJ software (scale bar = 50 μm) (values are mean ± SD, *n* = 4, *p* < 0.05 (*)).

**Figure 8:**
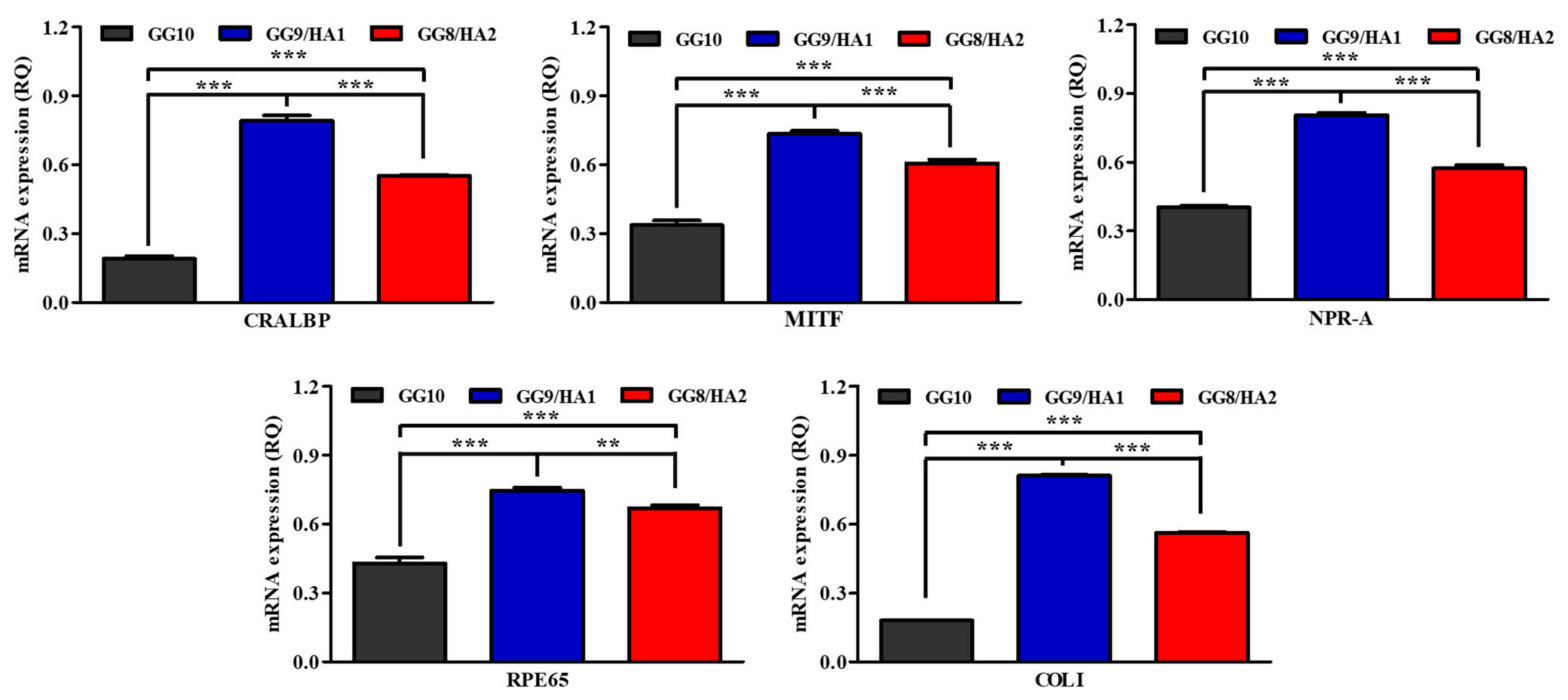
RPE-specific gene expression evaluated by RT-PCR with CRALBP, MITF, RPE65m NPR-A, and COL1 normalized by GAPDH (values are mean ± SD, *n* = 6, *p* < 0.01 (**), *p* < 0.001 (***)).

The immunofluorescence staining of RPE65, ZO-1, and phalloidin was carried out after 28 days of culture (Figure 7A). In particular, RPE-65 and ZO-1 are RPE-specific proteins. RPE-65 plays an important role in the cis–trans isomerization of retinol in the RPE-photoreceptor VA cycle, and it is a specific factor for RPE. ZO-1 was used to define the tight junction structure of the cultured RPE cells [53]. Phalloidin can be used to detect physical damage caused to RPE. Phalloidin staining clearly describes the cell membrane and serves to show the shape of the epithelium [54]. The expression intensity of the RPE-specific proteins was normalized by DAPI staining (Figure 7B). The GG groups showed some cells that did not express the RPE-specific protein (represented in yellow arrows) and showed the lowest expression level when compared to the GG/HA hydrogels. The GG9/HA1 and GG8/HA2 groups displayed all the cells expressing RPE-specific proteins. However, while GG9/HA1 showed cells with a round and healthy appearance, GG8/HA2 showed cells with a smaller and deformed shape, which may be due to the cell apoptosis from the poor mechanical and physicochemical properties. The gene expression of the encapsulated cells was analyzed after 28 days of culture by using RPE-specific genes, cellular retinaldehyde-binding protein (CRALBP), microphthalmia-associated transcription factor (MITF), natriuretic peptide receptor-A (NPR-A), and collagen type I (COL I) (Figure 8). CRALBP is involved in the regeneration of visual pigments and is one of the essential proteins for mature RPE [55]. NPR-A regulates gene expression associated with RPE cell proliferation or retinal fluid uptake [56]. COL I is involved in ECM formation and is one of the important components of RPE tissue [57]. MIFT is involved in the production of melanocytes and is required for normal RPE development [58]. The GG9/HA1 group exhibited the highest expression of RPE-related genes. In particular, genes that are related to the cell proliferation, growth, and structure of ECM exhibited a significant increase in the GG9/HA1 group, which may be due to the positive microenvironment and fast stress relaxation property. The above results confirm that the GG/HA complex can be applied as a promising biomaterial in RPE TE.

## 3. Materials and Methods

### 3.1. Preparation of GG/HA Hydrogel

HA powder (110 kDa, Bioland, Cheongju, Korea) was dissolved in distilled water (DW) at 4 °C at an amount of 1% until it was homogeneously mixed. The fabricated solution was stored at 4 °C to remove air bubbles. GG powder (Gelzan^TM^, Sigma-Aldrich, Burlington, MA, USA) was added to the DW at an amount of 1% (*w*/*v*) and stirred at 90 °C. After the GG solution was completely dissolved, the temperature was set to 40 °C. Then, the GG solution and HA solution were mixed at ratios of 10:0 = GG:HA, 9:1 = GG:HA, and 8:2 = GG:HA, which are named GG10, GG9/HA1, and GG8/HA2, respectively. After the mixture was thoroughly mixed, calcium chloride (CaCl_2_, SHOWA, TOKYO, Japan) was added to a total concentration of 0.2 mM and stirred for 20 min. Each solution was dispensed into a silicon mold of 3 mm height and 6 mm diameter and solidified by being placed at room temperature (RT) for 30 min.

### 3.2. Physicochemical Study 

#### 3.2.1. Mass Swelling Ratio (%)

The fabricated hydrogel samples were incubated in 1 mL of phosphate-buffered saline (PBS, pH 7.4, Gibco, Waltham, MA, USA) at 37 °C for 24 h. After 24 h, the PBS was removed, and the weight of the swelled hydrogels was measured (*W_i_*). Then, the samples were lyophilized, and the weight of the dried samples was recorded (*W_t_*). The mass swelling ratio (%) was calculated using following the Equation (1).

(1) $$\mathrm{Mass}\;\mathrm{swelling}\;\mathrm{ratio}\;(\%)\;=\;\frac{W_{t}}{W_{i}}\times 100\;\left(\%\right)$$

#### 3.2.2. Sol Fraction (%)

The sol fraction was studied by measuring the weight of freeze-dried hydrogel samples (*m_i_*). The samples were immersed in DW with slight agitation (80 rpm) for 1 h, and the samples were lyophilized. The dried weight of the samples was recorded (*m_f_*). The sol fraction (%) was calculated using following the Equation (2) [46].

(2) $$\mathrm{Sol}\;\mathrm{fraction}\;\mathrm{ratio}\;(\%)\;=\;\frac{m_{i}-m_{f}}{m_{i}}\times 100\;\left(\%\right)$$

#### 3.2.3. Weight Loss Ratio (%)

The weight loss ratio (%) was measured for 28 days. The initial weight of each sample (*W_i_*) was measured, and each sample was incubated in 1 mL PBS at 37 °C. Then, at specific time points (1, 7, 14, 21, and 28 days), the PBS was removed, and the hydrogel was blotted dry with filter paper. The weight of the hydrogel was recorded (*W_d_*), and the PBS was added again. The PBS was replaced every 3 days. The weight loss ratio (%) was calculated using Equation (3) [59].

(3) $$\mathrm{Weight}\;\mathrm{loss}\;(\%)\;=\;\frac{W_{i}-W_{d}}{W_{d}}\times 100\;\left(\%\right)$$

### 3.3. Mechanical Property Characterization

#### 3.3.1. Viscosity Evaluation

The viscosity, gelation temperature, and gelation time of the GG/HA hydrogel were measured using a viscometer (AMETEK Brookfield, Middleboro, MA, USA). For the viscosity and gelation temperature evaluation, the initial temperature of the viscometer was set at 37 °C, and 8 mL of the hydrogel solution was added in the viscometer. The router speed was set at 1 rpm, and cone and plate spindle (SC4-34 spindle, AMETEK Brookfield, Middleborough, MA, USA) were applied for this study. The temperature as gradually lowered until it reached 18 °C. The gelation time was measured at each gelation temperature of GG, GG9/HA1, and GG8/HA2. The router speed was set at 1 rpm, and cone and plate spindle were performed under the same conditions as the viscosity measurement. The temperature remained constant, and the time was measured at the point where the viscosity value increased abruptly.

#### 3.3.2. Injection Force Test

The injection force of the fabricated hydrogel was studied by following the previous reported study with a slight modification. The prepared samples were aspirated in a 1 mL syringe (Kovax-syringe, Korea Vaccine Co., Ltd., Seoul, Korea) with an amount of 500 μL. The syringes were capped with a $26G{\frac{1}{2}}^{\prime \prime }$ needle and stored at RT for 5 min to solidify the hydrogel solution. The samples were placed on a custom designed bracket, and the injection force test was carried out with a Texture Analyzer at a speed of 20 mm/min and a load cell of 20 N. The needle was submerged into the PBS solution to create a similar condition to that of an in vivo injection.

#### 3.3.3. Evaluation of Elastic Modulus and Relaxation Time

The initial elastic moduli and stress relaxation test was performed under an unconfined condition. The hydrogels were fabricated by following the method described in Section 3.1. The fabricated samples were incubated in PBS for 24 h. The prepared samples were placed on the cylinder plate of the Texture Analyzer (FTC, Sterting, VA, USA). The hydrogels were compressed at a speed of 1 mm/min with a load cell of 10 N. The height and diameter of the hydrogels were measured using a caliper (Mitutoyo, Gunpo, Korea) to evaluate the stress–strain curve. The slope of the first 5–10% strain was calculated to obtain the initial elastic modulus. For the stress relaxation analysis, the gel disks were compressed until the strain reached 20%. The strain was held constant, and the load was analyzed as a function of time. 

### 3.4. In Vitro Study

#### 3.4.1. Cell Culture 

ARPE-19 (ATCC^®^ CRL-2302^TM^, ATCC, Manassas, VA, USA) was cultured in Dulbecco’s modified Eagle medium/nutrient mixture F-12 (DMEM F-12, Gibco, Waltham, MA, USA), which was supplemented with 10% fetal bovine serum (FBS, Gibco, Waltham, MA, USA) and 1% penicillin/streptomycin (PS, Gibco, Waltham, MA, USA). The cells were incubated in a cell culture dish (Eppendorf, Framingham, MA, USA) at 37 °C and under 5% CO_2_ conditions. The cell culture medium was changed every 3 days, and the cells were subcultured until sufficient cells were obtained for further study.

#### 3.4.2. Cell Encapsulation within the Hydrogels

All the materials were sterilized prior to the cell encapsulation experiment. The hydrogel solution was fabricated by following the method described in the Section 3.1 and the hydrogel solution was filtered through a 0.45 μm filter (Sigma-Aldrich, Burlington, MA, USA). The temperature of the prepared hydrogel solution was maintained at 37 °C by storing the fabricated hydrogel solution in a water bath. The cultured ARPE-19 cells were trypsinized from the dish using 0.5% trypsin (Gibco, Waltham, MA, USA). The collected cells were co-cultured with the prepared hydrogel solution at a density of 5 × 10^6^ cells/mL at 37 °C. The cell-laden hydrogel solutions were poured into a Petri dish, and gelation was induced at RT for 5 min. The prepared cell-laden hydrogels were punched with a biopsy punch (Kai medical Biopsy Punch, Gifu, Japan) to make a cylindrical shape with a 6 mm diameter and 3 mm height. The cell-laden hydrogel samples were transferred into 24-well plates (Cell Culture Plate, STERILE, SPL Life Sciences Co., Ltd., Honshu, Korea). DMEM/F-12 media were added to each sample at an amount of 1 mL, and the plates were stored in standard humidified culture conditions (37 °C and 5% CO_2_). The culture medium was changed to a fresh solution every three days. 

#### 3.4.3. Morphological Analysis

A morphological analysis of the acellular and cell-laden hydrogels was conducted using a scanning electron microscope (BIO-LV SEM, Hitachi, SN-3000 Hitachi, Honshu, Japan). The acellular hydrogel samples were freeze-dried for the observation. The cell-laden hydrogels were fixed with 2.5% glutaraldehyde (Sigma-Aldrich, Burlington, MA, USA) for 24 h at 4 °C after 1 and 28 days of culture. The glutaraldehyde solution was removed and washed with PBS 3 times. The fixed cell-laden hydrogels were freeze-dried for dehydration. To evaluate the porous structure and morphology of the encapsulated cells, the freeze-dried samples were cross-sectioned and gold-sputtered using a vacuum sputter (SC500k, Emscope, Ashford, UK).

#### 3.4.4. Live/Dead Staining

A live/dead analysis was performed after 1 and 28 days of culture. A live/dead cell imaging kit (Invitrogen, Carlsbad, CA, USA) was used for this experiment according to the kit protocol. Briefly, calcein AM (green) and ethidium homodimer (red) were homogeneously mixed. The cultured cell-laden hydrogels were washed with PBS and then cut in half. The prepared samples were transferred to a cover glass bottom dish (SPL, lifescience, Pocheon, Korea), and the live/dead staining solution was added on top of the samples. The samples treated with the live/dead reagent were incubated at 37 °C with 5% CO_2_ for 30 min. The staining was observed under a super-resolution confocal laser scanning microscope (SR CLSM, LSM 880 with Airyscan, Carl Zeiss, Oberkochen, Germany) using the Z-stack method, and the height was fixed at 100 μm for all the samples. A quantitative study of the fluorescence intensity was performed using an image J program (1.49j version) [60].

#### 3.4.5. dsDNA Content

The cell-laden hydrogels were incubated at 37 °C and under 5% CO_2_ conditions for 1, 7, 14 and 28 days for the double-stranded DNA (dsDNA) content analysis. On specific days, the culture medium was removed, and the samples were washed with PBS 3 times. The weight of the samples was recorded, and the samples were stored at −60 °C until the entire culture study was finished. The study was conducted using a Quant-iT™ PicoGreen dsDNA Reagent kit (Invitrogen, Carlsbad, CA, USA) by following the manufacturer’s instructions. Briefly, the cell-laden hydrogels were homogenized in a 1X TE buffer with a glass tissue grinder (Wheaton, Primary, MN, USA), and further cell lysis was carried out using a freeze and thaw method. The sample solutions and Quanti-iT™ PicoGreen reagent were mixed at a ratio of 1:1 and placed in a black 96-well plate (Thermo Fisher Scientific, Waltham, MA, USA). The process was performed with protection from light. Standard curves were made with concentrations ranging from 0 to 2 ug/mL. The quantification was carried out using a microplate reader (EMax, Molecular Device, Sunnyvale, CA, USA) with the fluorescence intensities at an excitation wavelength of 485/20 nm and an emission wavelength of 528/20 nm.

#### 3.4.6. RT-PCR Analysis 

The cell-laden hydrogels were incubated in DMEM F-12 at 37 °C and under 5% CO_2_ conditions. After 28 days of culture, the samples were washed 3 times with PBS. For cell lysis, the samples were homogenized in 1 mL of Trizol (Takara, Tokyo, Japan) with the glass tissue grinder. Chloroform at an amount of 200 μL was added and homogeneously mixed with the samples. The samples were centrifuged at 15,000 rpm, at 4 °C, and for 15 min. After centrifugation, the transparent supernatant was transferred to a 1.5 mL Eppendorf tube (EP tube, Axygen, Union City, CA, USA), and isopropanol was subsequently added. The samples were incubated at 4 °C overnight. After incubating overnight, centrifugation was performed at 12,000 rpm, at 4 °C, and for 15 min. The supernatant was completely removed, and 75% ethanol was added to wash the samples. The centrifugation was performed at 7000 rpm, at 4 °C, and for 5 min. The supernatant was completely removed, and the gathered mRNA was diluted in Rnase-Dnase free water (Gibco, Waltham, MA, USA). The collected mRNA was quantified using a Biospectrophotometer (Eppendorf, Union City, CA USA). Expression markers and prepared mRNAs were added to TOPscript^TM^ One-step RT PCR DryMIX (Enzynomics, Daejeon, Korea) according to the kit protocol. cDNAs were synthesized and amplified using a PCR thermal cycler (Takara, Tokyo, Japan). The synthesized cDNAs were immersed in 0.5X Tris–Acetate–EDTA buffer (Takara, Tokyo, Japan) and then separated by electrophoresis (Takara, Tokyo, Japan) at 100 V on a 1% agarose gel visualized with ethidium bromide (EtBr, Sigma-Aldrich, Burlington, MA, USA). Collagen type I (COL I), cellular retinaldehyde (CRALBP), microphthalmia-associated transcription factor (MITF), atrial natriuretic peptide receptor (NPR-A), and retinal pigment epithelium-specific 65-kDa (RPE65) primers were used for the study [61]. All the genes were normalized by ß-actin, a housekeeping gene [62].

#### 3.4.7. Histological Analysis

Hematoxylin & Eosin (H&E) staining and fluorescence staining were observed after 1 and 28 days of culture. The hydrogels cultured for the specific days were fixed with 2.5% glutaraldehyde for 24 h at 4 °C. The glutaraldehyde solution was removed, and the samples were washed with PBS 3 times. The fixed samples were submerged in a cryomatrix (Thermo Fisher Scientific, Waltham, MA, USA) and frozen at −60 °C. The prepared samples were cryosectioned with a thickness of 7 μm using a cryomicrotome (Thermo Fisher Scientific, Waltham, MA, USA). The matrix was removed by washing with PBS 3 times, and staining was performed by following the standard staining procedure. Briefly, the samples were treated with Hematoxylin (Fisher Scientific, Hampton, NH, USA) for 5 min. The remaining residue was washed with PBS for 1 min and treated with Eosin (Sigma-Aldrich, Burlington, MA, USA) for 30 s. Dehydration was performed by subsequently treating with 70, 80, 90, and 100% ethanol (Samchun, Pyeongtaek, Korea) for 30 s. The stained samples were covered with malinol (MUTO PURE CHEMICALS. COC., LTD., Bunkyo-ku, Japan) and a cover glass to protect from deformation. Immunofluorescence staining was carried out by following the standard staining process. Briefly, the sectioned samples were washed with PBS and treated with 5% Bovine Serum Albumin (BSA, Sigma-Aldrich, Burlington, MA, USA) for 1 h to prevent nonspecific bonding. The primary antibodies RPE-65 (rabbit polyclonal, Thermo Fisher Scientific, Waltham, MA, USA) and ZO-1 (rat monoclonal, Santa Cruz Biotechnology, Piscataway, NJ, USA) were diluted at a ratio of 1:200 in 5% BSA solution and dispensed onto the samples, and the samples were stored at 4 °C overnight. After incubation, the solution was removed, and the samples were treated with the blocking solution for 10 min. The secondary antibodies Alexa Fluor 594 (rabbit anti-rat lgG (H+L), Invitrogen, Carlsbad, CA, USA), Alexa Flour 647 (goat anti-mouse lgG (H+L) highly crossed-adsorbed, Invitrogen, Carlsbad, CA, USA), and Alexa Fluor 488 phalloidin (goat anti-rabbit lgG (H+L) crossed-adsorbed Invitrogen, Carlsbad, CA, USA) were diluted in 5% BSA solution at a ratio of 1:500, and they were dispensed on the samples for 1 h at RT. The procedure was carried out under dark conditions. After 1 h of incubation, the samples were treated with a 4′, 6-Diamidene-2′-phenylindole dihydrochloride mounting (DAPI mounting, Ultra Cruz®,Mounting Medium with DAPI, Santa Cruz Biotechnology, Dallas, TX, USA, USA) and were covered with a cover glass. The edges were painted with nail polish to prevent the disappearance of the fluorescence.

#### 3.4.8. Statistical Analysis

All data are expressed as mean ± standard deviation (SD) values. GraphPad Prism 5.01 (GraphPad Software, La Jolla, CA, USA) was used for data analysis. A one-way ANOVA test was applied, and the differences were considered as *p* < 0.05 (*), *p* < 0.01 (**), and *p* < 0.001 (***).

## 4. Conclusions

In this study, injectable GG/HA hydrogels were proposed as effective biomaterials for the treatment of damaged retinas. Both GG and HA are known to be biocompatible substances. GG/HA hydrogels are manufactured using a simple physical crosslinking method without chemical modification. Hydrogels with appropriate amounts of HA showed improved physicochemical and mechanical properties suitable for injection into the retina. Moreover, the time-dependent stress relaxation property of the GG/HA hydrogel was enhanced when the appropriate amount of HA was loaded. In addition, the cellular compatibility of the GG/HA hydrogel in in vitro experiments was significantly improved in the fast-relaxing hydrogel. Cell encapsulation was successfully performed in the hydrogels, and the GG/HA hydrogel improved cell growth, viability, and adhesion. Compared to the pure GG hydrogel, the GG/HA hydrogel provided cells with an improved microenvironment for cellular activity. Moreover, mixing the appropriate amount of HA provided a higher expression of retinal-specific proteins and genes. Overall, these results demonstrate the remarkable potential of GG/HA hydrogels as an injectable hydrogel for retinal TE and the importance of the stress relaxation property when designing retinal TE hydrogels.

## Data Availability

Not applicable.

## Sample Availability

Samples of the compound are available from the authors.
